# Bacterial diversity associated with volatile compound accumulation in pit mud of Chinese strong-flavor baijiu pit

**DOI:** 10.1186/s13568-023-01508-z

**Published:** 2023-01-07

**Authors:** Yan Shoubao, Jia Yonglei, Zhang Qi, Pu Shunchang, Shi Cuie

**Affiliations:** 1 Department of biology engineering, Huainan Normal University, Huainan, 232038 Anhui China; 2 Department of biology and food engineering, Bozhou University, Bozhou, 236800 China; 3 Liquor Making Biotechnology and Application Key Laboratory of Sichuan Province, Sichuan University of Science & Engineering, Yibin, 644000 China

**Keywords:** Pit mud, Bacterial community, Volatile flavor compounds, Chinese strong-flavor Baijiu

## Abstract

Pit mud quality is a key parameter that impacts the quality of Chinese strong-flavor Baijiu production.This study was developed to explore spatial bacterial community distributions and the relationships between these distributions and the volatile compound accumulation within the pit mud used in the production of Chinese strong-flavor Baijiu. The results revealed *Lactobacillus pasteurii* and *Limnochorda pilosa* were found to be the dominant bacteria present in the upper wall, middle wall, and bottom pit mud layers, whereas the *Clostridium* genus was detectable at high levels in the lower layer of the pit wall and played a role in contributing to the overall aroma and flavor compounds in produced Chinese strong-flavor Baijiu, with *Clostridium* abundance being strongly correlated with caproic acid, ethyl caproate, ethyl butyrate, and hexanol levels as well as moderately correlated with butyric acid levels. The abundance of the *Lactobacillus* genus was positively correlated with levels of ethyl lactate, 1-butanol, and 2,3-butanediol. *Limnochorda pilosa* was closely associated with ethyl acetate levels. Additionally, the correlations between bacterial communities and chemical properties also investigated, and the results demonstrated PO4^3−^, total carbon, K^+^, humus, NH_4_^+^-N, and Mg^2+^ levels significantly affected the bacterial community structure of pit mud, and they were positively correlated with the relative abundance of *Clostridium*. Together, these findings can serve as a foundation for future studies exploring the mechanisms whereby volatile compounds accumulate in different pit mud layers, which facilitates the fermentation regulation and pit mud quality improvement of Chinese strong-flavor Baijiu.

**Supplementary Information:**

The online version contains supplementary material available at 10.1186/s13568-023-01508-z.

## Introduction

Chinese strong-flavor Baijiu is renowned for its characteristic “strong pit flavor, soft, sweet and mellow, harmonious flavor, and long aftertaste”, and it accounts for over 70% of total Baijiu sales in China (Yan et al. 2015). The aroma of Chinese strong-flavor Baijiu is primarily associated with the metabolic activities of the different microorganisms involved in its fermentation, with the dominant flavor of this type of Baijiu being derived from pit mud microorganisms. The pit mud used to ferment Chinese strong-flavor Baijiu is home to countless aromatic compound-producing microbes, including species of *Clostridium*, *Bacillus*, and *Methanobacter*, which play critical roles in determining the quality of the resultant Chinese strong-flavor Baijiu. During the fermentation process, the microbes that inhabit the pit mud interact with grains at the interface between these grains and the pit mud, with the resultant compound exchange leading to the production of various aromatic compounds (Yan et al. 2019), thus enabling these microbes to shape the taste and quality of this beverage. Traditionally, fermentation cellars are used for many years with the fermented grain placed in the lower portion of the pit cellar serving as a source of high-quality liquor. While the underlying mechanisms have yet to be fully clarified, position-dependent fermentation effects have been attributed to the distinct microbes that are primarily present within the lower portion of the pit cellar (Bi et al. 2022). To clarify how these microbes shape the process of Chinese strong-flavor Baijiu production, there is thus a clear need to examine microbial distributions and aroma compounds present in different spatial locations within the pit mud.

Levels of volatile compounds within pit mud serve as an important index by which the quality of the pit mud can be assessed, in addition to serving as a material basis for mutual exchange with fermented grains. Headspace solid-phase microextraction (HS-SPME) coupled with gas chromatography-mass spectrometry (GC-MS) is an east-to-conduct approach that has been widely used in recent years to characterize volatile compounds present within pit mud and grains used in distilling processes (Liu et al. 2017; Qian et al. 2021). Studies of the spatial distributions of aromatic compounds within fermented grains have revealed that the levels of esters and acids are higher in grain samples closer to the pit mud, thus demonstrating that the pit mud plays a role in the production of these volatile compounds (Tang et al. 2012). Pit mud organic acid content has also been shown to increase with cellar depth, indicating a significant difference in organic acid levels in different spatial positions within a given fermentation pit (Lei et al. 2020; Zhang et al. 2022) previously conducted quantitative and qualitative analyses of aromatic compounds present within pit mud and fermented grain sample, revealing a high degree of similarity between the dominant aromatic compounds in pit mud and grain samples and the relative positions of these dominant compounds. Further analyses have demonstrated that aromatic compound levels are higher in fermented grain samples from the sides of the fermentation pit relative to those in the central region, consistent with interactions and material exchange between the pit mud and these grains in the context of liquor fermentation.

Analyses of microbial communities in pit mud samples collected from different locations within fermentation pits (including the bottom of the cellar and the upper, middle, and lower portions of the cellar walls) have revealed marked differences in the spatial profiles of pit mud microbes (Hu et al. 2020). Pit mud physicochemical properties have been found to impact microbial survival, with water content, for example, influencing soil pH and microbial growth (Xiang et al. 2009). An appropriate microenvironmental pH can support alcohol fermentation while promoting the production of aromatic precursor compounds and other improvements in liquor quality (Liao et al. 2010). Ammonium nitrogen is required for the growth of microbes and the synthesis of a range of enzymes and other proteins, with appropriate ammonium nitrogen concentrations being critical to the maintenance of pit mud quality and the overall improvement of liquor quality (Hu et al. 2021). Both phosphorus and potassium in pit mud can also support microbial growth (Wu et al. 2022). With rising pit age, increases in water, organic matter, and available potassium concentrations have been reported (Li et al. 2018). The ability of pit mud to impact the produced Chinese strong-flavor liquor is also related to the `flavor compounds present within the pit mud. Indeed, several flavor compounds have been linked to improvements in Chinese strong-flavor liquor quality and flavor, with the types and levels of these aromatic compounds being related to the aging-related pit microbial community structures (Wu et al. 2022), and to spatial locations within fermentation pits (Hu et al. 2020). To date, several studies have explored microbial communities present within pit mud from fermentation cellars of different ages, whereas relatively few studies have examined the spatial distributions of bacterial communities and the relationships between these distributions and volatile compound accumulation in pit mud used to produce Chinese strong-flavor liquor.

To date, several studies have explored microbial communities present within pit mud from fermentation cellars of different ages, whereas relatively few studies have examined the spatial distributions of bacterial communities and the relationships between these distributions and volatile compound accumulation in pit mud used to produce Chinese strong-flavor liquor.

Previous studies concerning microbial community structure have been performed based on culture-dependent methods. However, most of the microorganisms of pit mud are uncultured or difficult to culture, and culture-dependent method is difficult to reveal the inner pattern comprehensively and objectively. In contrast, molecular biology approaches have been proven to be powerful tools in providing a more complete inventory of the microbial diversity in environmental samples (Wang et al. 2011). By the analysis of the bands migrating separately on the DGGE gels, polymerase chain reaction denaturing gradient gel electrophoresis (PCR-DGGE) has succeeded in obtaining phylogenetic information about the microorganisms existing fields of environmental ecology and microbiology (Deng et al. 2012), thus, limitations of the traditional culture method can be avoided, and the original status of the microbial community in the pit mud can be accurately reflected.

The present study was thus developed in an effort to explore differences in the bacterial communities present in different spatial positions in the pit mud of a fermentation pit used in the production of Chinese strong-flavor liquor. Additionally, an HS-SPME-GC-MS approach was used to evaluate volatile compound fingerprints in these different spatial locations, while correlation analyses were used to examine relationships between pit mud physicochemical properties, bacterial community composition, and volatile aromatic compound levels as a means of clarifying the spatial dynamics of microbial communities and volatile compound production within pit mud.

## Materials and methods

### Pit mud sample collection

The samples of pit mud were collected on November 18, 2021 from a 20-year-old fermentation pit in a strong-flavor liquor distillery in Anhui Province, China. These samples were collected from four positions within the fermentation pit, including the upper, middle, and lower layers of the cellar wall as well as the bottom of the pit. Sample plots were divided into 8 subplots (center and edges), with the exception of the bottom layer which was separated into 9 subplots (the side center, side edges, and bottom middle). Approximately 100 g of pit mud was collected from each subplot with a sterile hollow cylindrical sampler (Puluody, China) to a depth of ~ 5 cm. Samples were then thoroughly mixed and stored at −20 °C in sterile polyethylene bags prior to analysis.

### Eubacterial community analyses

#### DNA extraction and PCR amplification

A total of 1 g of pit mud per sample was mixed for 5 min in 25 mL of phosphate buffered saline (PBS) (0.1 mol/L, pH 8.0), followed by centrifugation for 10 min at 600 xg at 4 °C. Pellets were then rinsed three times with PBS, followed by centrifugation at 12,000 xg for 10 min at 4 °C. The pellet was then washed three times using PBS, followed by storage at −20 °C. Samples were prepared in triplicate. Genomic DNA was isolated from these samples with a Soil Genomic DNA Rapid Extraction Kit (Omega) based on provided directions, after which samples were analyzed via 1% (w/v) agarose gel electrophoresis and stored at −20 °C.

The PCR-mediated amplification of bacterial 16 S rRNA sequences was initially performed using the 27 F/1492R primers, after which nested PCR was performed with the DGGE primers GC-338 F/518R to yield amplified V3 16 S rRNA sequences for DGGE analysis. All PCR reactions were performed in a 50 µL volume containing 5 µL 10×PCR buffer, 3.2 µL dNTP Mixture (2.5 mM), 0.4 µL of Premix ExTaq (5 U/µL), 1 µL of each primer (20 µM), 50 ng of template DNA, and double-distilled water (ddH_2_O) to a final volume of 50 µL.

The thermocycler program was as follows: 94 °C for 5 min, followed by 30 cycles of 94 °C for 60 s, 55 °C for 45 s, decreasing by 0.5 °C/cycle, and 72 °C for 1 min. Samples were then processed through 15 cycles of 94 °C for 45 s, 55 °C for 45 s, 72 °C for 1 min, and a final extension at 72 °C for 5 min prior to holding at 16 °C. The amplified products were analyzed via 1% agarose gel electrophoresis.

#### PCR-DGGE analyses

Denaturing gradinent electrophoresis (DGGE) analyses were performed using a Dcode system instrument (Bio-Rad). Briefly, PCR products were applied to 7% polyacrylamide gels in 1×Tris acetate-EDTA buffer (TAE) running buffer. DGGE analyses were performed using a denaturing gradient with denaturant (7 M urea and 40% formamide) concentrations from 35 to 55%. Gels were separated for 5 h at 150 V 60 °C, after which they were subjected to silver staining for 15 min (Yan et al. 2019). Gels were then imaged with a Bio-Rad Gel Doc XR scanner (Bio-Rad, USA), and bands were selected, excised with a clean blade, and eluted by incubating them overnight in 30 µL of sterile distilled water at 4 °C to permit DNA diffusion. Samples were then stored at −20 °C.

#### DGGE profile sequence analyses

Eluted samples of DNA were re-amplified with the same PCR settings and GC-clamp primers as used above, after which these products were analyzed via DGGE with the sample DNA samples used in the initial analysis to enhance purity and to confirm sequence identity. Bands in the same position as those selected above were excised and eluted using the same methods. Followed by re-amplification with the same primers without the GC-clamp. Purified DNA samples were introduced into the pMD18-T vector (Tiangen) and sequenced by Sangon (Shanghai, China). The BLAST tool was then used to search GenBank for these sequences to identify the closest known relatives for the obtained partial 16 S rRNA sequences.

### Volatile compound analyses

Volatile acids were determined by distillation extraction-gas chromatography (Yan et al. 2015). A 100 g pit mud sample and 200 mL of 60% (v/v) ethanol were transferred into 500 mL round-bottom flask, the flask was heated and a 100 mL solution was distilled from the mixture. The obtained solution was analysed using a gas chromatograph according to our previous reports (Yan et al. 2015).

As for other volatile compounds (esters, alcohols, aldehydes, ketones, alkanes, and volatile phenols), they were extracted using a solid-phase microextraction extraction (SPME) head (DVB-CAR-PDMS). Briefly, 1 g pit mud samples were added to 10 mL bottles to which 1 mL of water, 0.25 g NaCl, and 100 µL of 2-octanol (70 mg/L, internal standard) were added. The mixture was then incubated for 30 min in a 50 °C water bath. The DVB-CAR-PDMS fiber of SPME was then exposed to the bottle headspace 2.0 cm from the surface of the liquid for 30 min. Once extraction was complete, this fiber was introduced into the GC injector for 5 min for thermal desorption, with desorbed volatile compounds then being analyzed and characterized.

A GC-MS instrument (Agilent 6890 GC and Agilent 5975 mass selective detector (MSD); Agilent, CA, USA) was used to separate and analyze volatile compounds in these extracts with the following settings: DB-Wax column (Length of 60 m, 0.25 mm internal diameter, 0.25 μm film thickness); carrier gas: helium; flow rate: 1 mL/min; split ratio 5:1; inlet temperature: 250 °C. The oven temperature programmed from 40 °C (held 2 min), ramped at 5 °C/min to 80 °C (held for 2 min), then the temperature was increased to 230 °C at a rate of 7 °C per minute, and maintain for 8 min. MS analyses were performed with an electrospray ionization source with a 70 eV electron energy, an ion source temperature of 230 °C, and a scanning range of 28–500 amu in full scan mode. Samples were assessed in triplicate, with quantitative and qualitative analyses being conducted as reported previously by Yan et al. (2019).

### Analyses of pit mud physicochemical properties

Pit mud moisture levels were detected via a gravimetric approach by drying samples for a minimum of 6 h at 105 °C. Pit mud pH was assessed using a pH meter and a 1:10 pit mud to boiled deionized water sample. Ammonium (NH_4_^+^-N) present within pit mud was extracted using 10% (w/v) NaCl and measured with a UV–vis spectrophotometer (UV-9000, Bejing Puxi Instruments Co., Ltd). Total carbon levels in air-dried powdered pit mud samples were assessed with an Elementar instrument (Shanghai Yuanxi TOC-5000, China) in CHNS mode with the burning tube being heated to 1150 °C and the reducing tube being heated to 850 °C. Pure water was used to extract K^+^, PO_4_^3−^, soluble Mg^2+^, and soluble Ca^2+^ from air-dried pit mud samples at a 1:10 (w/v) ratio, after which their concentrations were analyzed with an ion chromatograph (ICS5000+, ThermoFisher) equipped with conductivity detector (ICS-5000 + -DC) and a CS12 column (IonPac, ThermoFisher, 4 mm × 250 mm). A 25 µL injection volume was used for all analyses, with methane sulfonic acid (20 mM) as the carrier fluid at a 1 mL/min flow rate, and a constant column temperature of 30 °C.

## Results

### DGGE-based bacterial community characterization

**Fig. 1:**
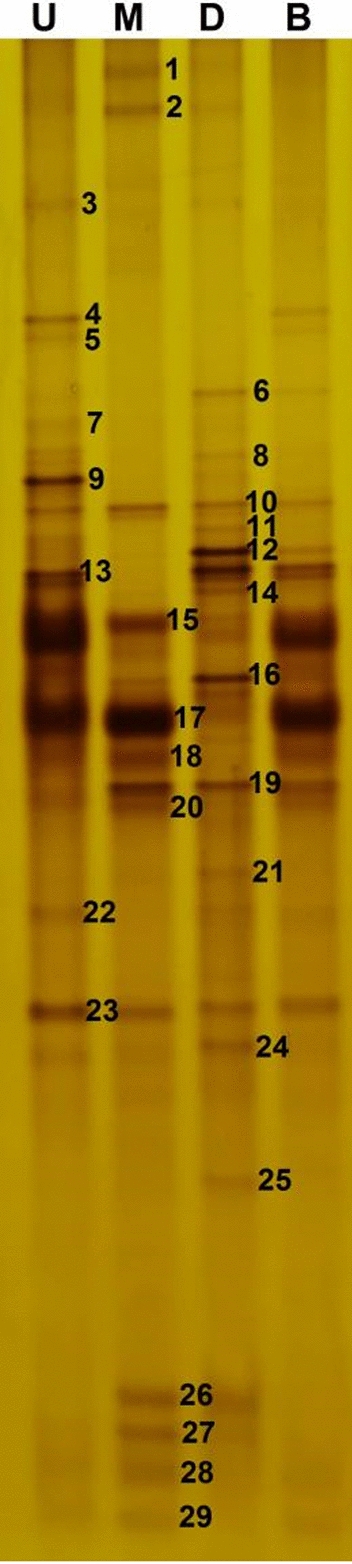
PCR-DGGE fingerprints based on 16 S rRNA genes amplified from bacteria found in pit mud samples collected from different positions within the fermentation pit. Lanes U, M, D, and B respectively correspond to pit mud samples from the upper wall, middle wall, lower wall, and bottom layers of the cellar. Bands marked by numbers were excised for sequencing, with the resultant alignment results being compiled in Table 2

Initially, a DGGE fingerprinting approach was used to characterize the bacterial community profiles in different pit mud samples from the upper, middle, and lower layers of the pit wall and from the bottom of the pit (Fig. 1). There were notable differences in the microbial composition of pit mud samples from these different spatial locations, with species richness being sufficient to effectively separate these four samples while revealing that the samples from the lower layer of the pit wall exhibited the greatest number of bands (18), followed by samples from the middle and upper pit mud wall layers (15) (Table 1). There were no significant differences in evenness index values for these different pit mud samples, suggesting that they all exhibit largely homogeneous ecosystems. Notably, the Shannon-Wiener index values for samples from the lower layer of the pit mud wall were highest (3.42) in these PCR-DGGE profiles, confirming the presence of a high number of different species of bacteria in these samples.

**Table 1:** Bacterial diversity indices in pit mud samples as calculated based on the DGGE banding patterns shown in Fig. 1

	Shannon-Wiener	Evenness	Richness
1	3.2	0.996	15
2	2.98	0.997	15
3	3.42	0.997	18
4	3.07	0.995	13

**Table 2:** BLAST Identified gene sequences of 16 S rDNA - derived bands excised from a DGGE gel

Band no.^a^	Closest relative (NCBI accession no.)	Identity (%)^b^
1	*Caloramator mitchellensis* (NR_117542.1 )	98.98
2	*Janthinobacterium lividum* (NR_026365.1)	100.00
3	*Tepidanaerobacter acetatoxydans* (NR_074537.1)	97.04
4	*Frondibacter aureus* (NR_134733.1)	97.83
5	*Syntrophomonas curvata* (NR_025752.1)	96.33
6	*Petrimonas sulfuriphila* (NR_042987.1)	100.00
7	*Lutaonella thermophila* (NR_044451.1)	95.65
8	*Thermoclostridium caenicola* (NR_126170.1)	95.29
9	*Hydrogenoanaerobacterium saccharovorans* (NR_044425.1)	95.88
10	*Clostridium sporosphaeroides* (NR_044835.2)	98.22
11	*Sedimentibacter hydroxybenzoicus* (NR_029146.1)	96.51
12	*Clostridium kluyveri* (NR_074447.1)	97.63
13	*Petrimonas mucosa* (NR_148808.1)	96.65
14	*Proteiniphilum saccharofermentans* (NR_148807.1)	96.24
15	*Lactobacillus pasteurii* (NR_117058.1)	96.41
16	*Clostridium luticellarii* (NR_145907.1)	100.00
17	*Limnochorda pilosa* (NR_136767.1)	96.18
18	*Phocea massiliensis* (NR_144748.1)	97.90
19	*Fermentimonas caenicola* (NR_148809.1)	98.19
20	*Proteiniphilum acetatigenes* (NR_043154.1)	100.00
21	*Anaeromassilibacillus senegalensis* (NR_144727.1)	96.38
22	*Lactobacillus acetotolerans* (NR_044699.2)	100.00
23	*Christensenella massiliensis* (NR_144742.1)	96.52
24	*Clostridium jeddahense* (NR_144697.1)	98.03
25	*Clostridium limosum* (LC036318.1)	98.51
26	*Ardenticatena maritima* (NR_113219.1)	98.88
27	*Syntrophaceticus schinkii* (NR_116297.1)	96.34
28	*Pelomonas puraquae* (JQ660112.1)	96.33
29	*Atopobium rimae* (NR_113038.1)	97.53

Bacterial DGGE pattern for amplified 16 S rDNA V3 gene fragments revealed 15, 15, 18, and 13 bands in the upper wall, middle wall, lower wall, and bottom pit mud layers, respectively, with lower wall samples thus exhibiting more DGGE bands than other samples. In total, 29 bands were excised for sequencing, leading to the identification of 24 families of *Caloramator*, *Janthinobacterium*, *Tepidanaerobacter*, *Frondibacter*, *Clostridium*, *Petrimonas*, *Lutaonella*, *Hydrogenoanaerobacterium*, *Thermoclostridium*, *Sedimentibacter*, *Syntrophomonas*, *Petrimonas*, *Proteiniphilum*, *Lactobacillus*, *Limnochorda*, *Phocea*, *Fermentimonas*, *Proteiniphilum*, *Anaeromassilibacillus*, *Christensenella*, *Ardenticatena*, *Syntrophaceticus*, *Pelomonas*, and *Atopobium* (Table 2, Additional file 1). *Lactobacillus pasteurii* (Band 15) and *Limnochorda pilosa* (Band 17) were found to be the dominant bacteria present in the upper wall, middle wall, and bottom pit mud layers, whereas *Clostridium kluyveri* (Band 12) and *Clostridium luticellarii* (Band 16) exhibited the opposite trends and were only present in the lower wall pit mud samples. *Lactobacillus pasteurii* (Band 15) and *Lactobacillus acetotolerans* (Band 22) were the primary lactic acid bacteria species identified in these samples, while *Thermoclostridium caenicola* (Band 8), *Clostridium sporosphaeroides* (Band 10), *Clostridium kluyveri* (Band 12), *Clostridium luticellarii* (Band 16), *Clostridium jeddahense* (Band 24), and *Clostridium limosum* (Band 25) were the main Clostridium species found in these different pit mud layers.

### Analysis of spatial pit mud volatile compound profiles

Next, the concentrations and retention times (in minutes) for different volatile compounds found in these pit mud samples were analyzed (Table 3). In total, 53 volatile compounds were identified and quantified, including 15 acids, 13 esters, 9 alcohols, 6 aldehydes, 3 ketones, 4 alkanes, and 3 volatile phenols. Marked differences in the distributions of these volatile compounds were observed across spatial locations.

**Table 3:** The volatile aroma compounds detected and measured in the samples collected from different spatial positions of pit

Number	Aroma compounds	Retention time (min)	Identification	Contents of volatile aroma compounds of pit mud(µg/mg)			
				U	M	D	B
*Volatile acids*							
AC1	Propionic acid	12.383	MS, RI	69.929 ± 2.214	56.783 ± 1.254	34.543 ± 1.987	6.254 ± 0.587
AC2	Butyric acid	14.547	MS, RI	135.802 ± 5.365	358.306 ± 6.321	469.598 ± 4.025	364.421 ± 3.587
AC3	Pentanoic acid	16.761	MS, RI	ND	12.452 ± 0.584	87.452 ± 5.026	79.444 ± 1.357
AC4	2-Benzylpropionic acid	17.746	MS, RI	ND	ND	27.673 ± 1.025	38.215 ± 3.541
AC5	4-methyl-pentanoic acid	18.351	MS, RI	ND	5.353 ± 0.325	13.488 ± 0.897	9.967 ± 0.869
AC6	Caproic acid	19.468	MS, RI	308.398 ± 12.321	783.231 ± 16.214	2421.936 ± 16.321	1652.168 ± 10.214
AC7	Heptanoic acid	21.72	MS, RI	328.312 ± 6.325	210.432 ± 4.321	95.562 ± 3.351	ND
AC8	Octanol acid	24.193	MS, RI	638.129 ± 8.369	907.256 ± 3.651	1006.955 ± 8.354	1142.957 ± 9.325
AC9	Nonanoic acid	26.735	MS, RI	24.469 ± 2.351	35.4226 ± 2.584	45.544 ± 2.102	20.342 ± 0.156
AC10	n-Decanoic acid	28.514	MS, RI	27.159 ± 1.695	61.789 ± 1.587	77.172 ± 1.650	29.683 ± 0.365
AC11	Benzoic acid	30.35	MS, RI	9.905 ± 0.895	22.745 ± 1. 021	40.110 ± 1.365	22.793 ± 0.201
AC12	Decanoic acid	31.025	MS, RI	ND	ND	12.919 ± 0.987	6.754 ± 0.965
AC13	Benzeneacetic acid	31.675	MS, RI	ND	21.556 ± 0.202	62.141 ± 2.036	15.169 ± 1.589
AC14	Tetradecanoic acid	32.956	MS, RI	ND	ND	14.0171 ± 0.876	8.432 ± 0.968
AC15	n-Hexadecanoic acid	34.615	MS, RI	ND	7.338 ± 0.658	8.357 ± 0.879	7.060 ± 0.756
	Σ			1542.103 ± 16.325	2482.6636 ± 12.354	4417.4671 ± 25. 362	3403.659 ± 21.021
*Esters*							
ES1	Ethyl valerate	2.176	MS, RI	ND	9.751 ± 0.768	41.516 ± 1.036	20.145 ± 1.879
ES2	Ethyl acetate	3.274	MS, RI	320.23 ± 7.558	516.276 ± 23.258	833.687 ± 22.369	670.564 ± 21.527
ES3	Ethyl caproate	4.012	MS, RI	210.21 ± 6.321	1102.194 ± 20.021	1534.301 ± 18.654	1210.365 ± 18.654
ES4	Ethyl butyrate	6.125	MS, RI	118.356 ± 8.943	469.735 ± 26.793	632.441 ± 21.786	535.436 ± 20.894
ES5	Ethyl heptanoate	6.529	MS, RI	218.620 ± 8.32	243.079 ± 2.065	428.677 ± 10.365	321.845 ± 9.587
ES6	Ethyl benzoate	9.140	MS, RI	17.566 ± 0.987	24.534 ± 1.231	48.611 ± 0.986	36.397 ± 2.654
ES7	Ethyl nonanoate	12.301	MS, RI	11.324 ± 1.032	19.631 ± 1.032	28.086 ± 0.986	23.254 ± 1.841
ES8	Ethyl lactate	12.418	MS, RI	991.863 ± 27.32	787.467 ± 11.236	357.324 ± 10.587	465.320 ± 21.695
ES9	Ethyl caprate	14.137	MS, RI	ND	47.389 ± 2.365	230.889 ± 6.354	118.558 ± 1.005
ES10	Ethyl undecanoate	17.569	MS, RI	ND	7.167 ± 0.865	22.000 ± 0.864	10.235 ± 0.986
ES11	Benzeneacetic acid	18.099	MS, RI	ND	44.595 ± 1.036	63.735 ± 1.032	46.626 ± 0.653
ES12	Ethyl 3-phenylpropanoate	19.990	MS, RI	52.648 ± 1.365	70.878 ± 2.351	88.514 ± 4.235	80.603 ± 4.658
ES13	Ethyl tridecanoate	20.559	MS, RI	12.576 ± 0.989	9.563 ± 0.897	ND	ND
ES14	Ethyl phenylacetate	22.372	MS, RI	ND	ND	9.684 ± 0.945	6.626 ± 0.876
ES15	Ethyl myristate	26.401	MS, RI	ND	7.743 ± 0.852	23.838 ± 3.621	19.523 ± 1.894
ES16	Ethyl Palmitate	28.212	MS, RI	31.831 ± 3.025	114.508 ± 6.321	157.149 ± 9.362	183.678 ± 4.658
	Σ	1985.224	3474.51	1985.224 ± 8.980	3474.51 ± 2.125	4500.452 ± 1111	3789.175 ± 17.879
*Alcohols*							
AL1	Hexanol	7.28	MS, RI	5.211 ± 0.421	11.036 ± 1.982	27.368 ± 3.659	16.124 ± 2.033
AL2	Enanthol	11.241	MS, RI	6.263 ± 0.206	3.955 ± 0.057	2.775 ± 0.082	1.187 ± 0.032
AL3	Isobutanol	11.342	MS, RI	16 0.215 ± 0.596	9.356 ± 0.163	7.625 ± 0.355	4.845 ± 0.085
AL4	Isooctanol	12.105	MS, RI	8.389 ± 0.732	4.279 ± 0.389	2.207 ± 0.303	0.875 ± 0.069
AL5	1-Butanol	12.675	MS, RI	0.786 ± 0.462	4.268 ± 0.863	7.441 ± 0.986	5.079 ± 0.458
AL6	2,3-butanediol	13.432	MS, RI	ND	1.487 ± 0.354	3.096 ± 0.855	2.0154 ± 0.019
AL7	1-Pentanol	15.090	MS, RI	0.897 ± 0.101	3.653 ± 0.563	6.143 ± 0.996	5.321 ± 0.686
AL8	1-nonanol	15.966	MS, RI	7.232 ± 0.203	6.167 ± 0.202	3.147 ± 0.303	2.847 ± 0.303
AL9	2-Heptanol	16.620	MS, RI	3.827 ± 0.682	2.565 ± 0.063	1.897 ± 0.063	1.028 ± 0.013
	Σ			32.605 ± 1.641	35.730 ± 2.036	61.699 ± 4.233	39.321 ± 1.032
*Aldehydes*							
AD1	2-Heptenal	7.879	MS, RI	4.715 ± 0.398	3.267 ± 0.368	1.132 ± 0.132	0.587 ± 0.0498
AD2	Nonaldehyde	9.453	MS, RI	0.765 ± 0.056	3.188 ± 0.296	6.132 ± 0.568	4.012 ± 0.365
AD3	Benzaldehyde	12.809	MS, RI	1.315 ± 0.980	2.332 ± 0.205	4.366 ± 0.396	3.254 ± 0.296
AD4	2-undecenal	17.723	MS, RI	0.875 ± 0.056	1.925 ± 0.123	3.135 ± 0.268	2.015 ± 0.158
AD5	Pentanal	18.202	MS, RI	0.765 ± 0.063	1.897 ± 0.106	2.278 ± 0.260	1.968 ± 0.103
AD6	2-phenyl-2-butenal	21.621	MS, RI	1.032 ± 0.012	1.378 ± 0.101	4.543 ± 0.385	2.014 ± 0.186
	Σ			9.467 ± 0.851	13.987 ± 0.998	21.586 ± 0.968	13.85 ± 1.103
*Ketones*							
KE1	2-octanone	6.576	MS, RI	8.419 ± 0.098	3.251 ± 0.299	2.014 ± 0.203	ND
KE2	2-Heptanone	9.354	MS, RI	13.902 ± 0.986	8.564 ± 0.787	3.214 ± 0.654	0.987 ± 0.088
KE3	Undecanone	17.689	MS, RI	3.564 ± 0.336	8.572 ± 0.796	13.715 ± 1.021	9.254 ± 0.087
	Σ			25.885 ± 1.035	20.387 ± 0.212	18.943 ± 1.036	10.241 ± 0.098
	*Alkanes*		MS, RI				
AK1	Dodecamethylcyclohexasiloxane	7.784	MS, RI	6.269 ± 0.556	8.251 ± 0.774	14.340 ± 0.985	10.254 ± 1.213
AK2	Decamethylcyclopentasiloxane	2.239	MS, RI	24.768 ± 0.205	12.354 ± 0.105	8.657 ± 0.754	2.587 ± 0.302
AK3	Decamethyltetrasiloxane	19.247	MS, RI	23.010 ± 0.271	11.587 ± 0.989	6.587 ± 0.563	3.254 ± 0.289
AK4	2-phenyl-2-butenal	21.556	MS, RI	ND	9.564 ± 0.785	ND	ND
	Σ			54.047 ± 4.058	41.756 ± 0.687	29.584 ± 0.587	16.095 ± 0.989
*Volatile phenols*							
VP1	Phenol	24.562	MS, RI	ND	1.871 ± 0.152	3.254 ± 0.285	2.012 ± 0.156
VP2	2-Methylphenol	24.603	MS, RI	27.519 ± 1.996	35.602 ± 2.698	28.161 ± 1.365	32.985 ± 2.032
VP3	3-methylphenol	24.799	MS, RI	10.701 ± 1.023	ND	ND	ND
	Σ			22.22 ± 1.354	37.473 ± 2.654	28.415 ± 2.023	21.997 ± 1.652

Acids were the most abundant and important aroma compounds in these pit mud samples (Table 3). Twelve acids were present at the highest levels in the lower wall pit mud samples (Butyric acid, pentanoic acid, 2-benzylpropionic acid, 4-methyl-pentanoic acid, caproic acid, nonanoic acid, n-decanoic acid, benzoic acid, decanoic acid, benzeneacetic acid, and tetradecanoic acid), with caproic acid (2421.936 ± 16.321 µg/mg) and butyric acid (469.598 ± 4.025 µg/mg) being the most abundant in these samples. The content of propionic acid (69.929 ± 2.214 µg/mg) was the highest in up layer of pit mud, followed by samples from the middle and lower wall layers. Samples from the bottom of the pit exhibited the highest levels of 2-benzylpropionic acid (38.215 ± 3.541 µg/mg) and octanol acid (1142.957 ± 9.325 µg/mg) relative to other analyzed spatial positions.

The results of this study suggest that ester levels were higher in the lower wall and bottom pit mud layers relative to the upper wall and middle wall layers. The greatest variety of esters was also observed in pit mud samples from the lower wall and bottom of the fermentation pit (Table 3), with the exception of ethyl tridecanoatel, which was uniquely present in the upper and middle wall layers. The highest concentrations of ethyl valerate, ethyl hexanoate, ethyl heptanoate, ethyl benzoate, ethyl nonanoate, ethyl caprate, ethyl undecanoate, benzeneacetic acid, ethyl 3-phenylpropanoate, ethyl phenylacetate, and ethyl myristate were observed in the lower wall pit mud layer, with ethyl hexanoate, ethyl heptanoate, and ethyl caprate levels being significantly higher than those of other esters, reaching average concentrations of up to 912.754 ± 9.185 µg/mg. This was particularly true for ethyl hexanoate, which is a predominant flavor compound in Chinese strong-flavor liquor, and accounted for over 70% of the total ester content. Maximal ethyl palmitate levels were observed in the bottom pit mud layer.

Alcohols are also the group with comparative high content of volatile composition in pit mud. Total alcohol content was found to be highest in the lower wall pit mud layer (61.699 ± 4.233 µg/mg), followed by the bottom layer (39.321 ± 1.032 µg/mg). Of the detected alcohols, enanthol, isobutanol, isooctanol, and 2-heptanol were present at the highest levels in the lower wall pit mud layer, whereas the highest levels of enanthol, isobutanol, isooctanol, and 2-heptanol were detected in the upper wall pit mud layer.

Six different aldehydes were detected in all pit mud layer samples, among which nonaldehyde, benzaldehyde, 2-undecenal, pentanal, and 2-phenyl-2-butenal were detected at the highest levels in the lower wall pit mud samples, whereas 2-heptenal exhibited the opposite trend, being present at the highest levels in the upper pit mud layer.

Total ketone and alkane levels were highest in the upper wall pit mud layer, while maximum total volatile phenol levels were observed in the middle wall pit mud layer.

### Pit mud physicochemical properties

The physicochemical properties of samples of pit mud collected from different spatial positions are compiled in Table 4. There were clear increasing trends in the levels of moisture, pH, and Ca^2+^ in pit mud samples from the upper layer to the deepest layer of the pit corresponding to a physicochemical gradient within the fermentation pit. The K^+^ content in the bottom layer of pit mud was more similar to that in the lower layer of the pit mud wall and significantly higher than that in other pit mud samples, whereas maximum NH_4_^+^-N, total carbon, humus, Mg^2+^, and PO_4_^3−^ levels were observed in the lower wall pit mud layer followed by the bottom and middle pit mud layers.

**Table 4:** Pit mud physicochemical properties for samples collected from different spatial positions

Parameter	U	M	D	B
Moisture (%)	30.68 ± 2.87	34.18 ± 2.42	37.68 ± 2.58	39.32 ± 2.33
pH	5.28 ± 0.36	6.09 ± 0.25	7.30 ± 0.49	9.01 ± 0.87
NH_4_^+^-N (g/kg)	2.16 ± 0.19	3.77 ± 0.28	5.13 ± 0.29	4.19 ± 0.31
Total carbon (%, ads)	1.17 ± 1.29	1.32 ± 0.19	1.78 ± 0.22	1.58 ± 0.29
Humus (%, ads)	5.50 ± 0.21	9.121 ± 0.68	16.31 ± 0.89	13.58 ± 1.29
PO4^3−^ (mg/kg, ads)	195.28 ± 17.16	246.37 ± 18.26	385.19 ± 20.27	294.41 ± 19.38
K^+^ (mg/kg, ads)	528.29 ± 45.69	721.60 ± 31.17	1127.45 ± 48.75	1123.57 ± 80.13
Mg^2+^ (mg/kg, ads)	143.61 ± 56.22	186.51 ± 41.70	251.56 ± 47.10	215.70 ± 62.36
Ca^2+^ (mg/kg, ads)	359.31 ± 15.39	437.48 ± 20.37	509.78 ± 22.25	708.22 ± 43.22

(1) ads means air-dry samples. (2) U, M, D, and B respectively represent pit mud samples collected from up wall layer of cellar, middle wall layer of cellar, down wall layer of cellar, and bottom layer of cellar, and were sampled from the same fermentation cellar. (3) All data are presented as means ± standard deviations

### Correlations between bacterial communities and chemical properties

**Fig. 2:**
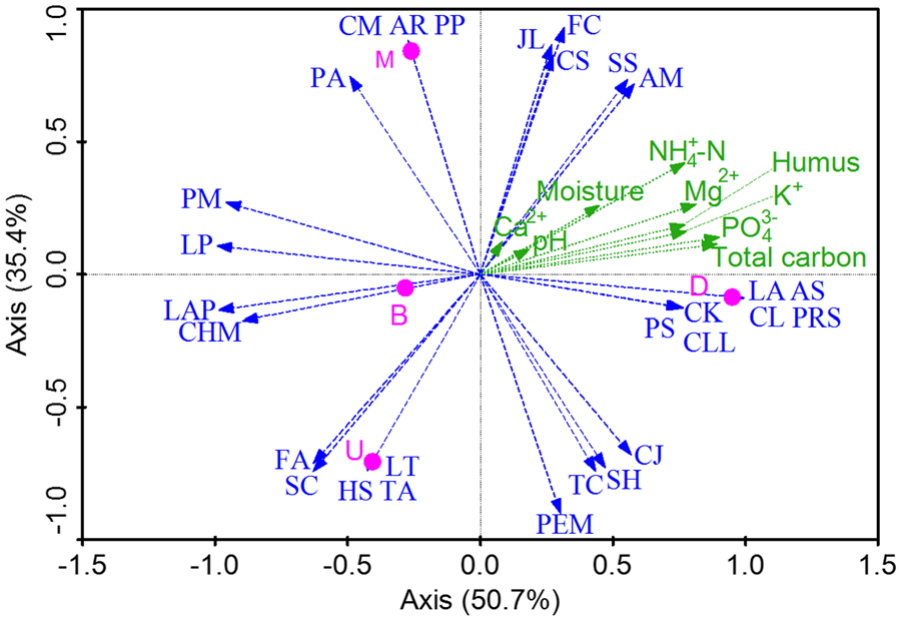
Redundancy analysis (RDA) of bacterial communities and chemical properties. Arrows denote the magnitude and directionality of biogeochemical attributes associated with microbial community structure. CM: *Caloramator mitchellensis*, JL: *Janthinobacterium lividum*, FA: *Frondibacter aureus*, SC: *Syntrophomonas curvata*, PS: *Petrimonas sulfuriphila*, LT: *Lutaonella thermophila*, HS: *Hydrogenoanaerobacterium saccharovorans*, TC: *Thermoclostridium caenicola*, CS: *Clostridium sporosphaeroides*, SH: *Sedimentibacter hydroxybenzoicus*, CK: *Clostridium kluyveri*, PEM: *Petrimonas mucosa*, PRS: *Proteiniphilum saccharofermenta*, LAP: *Lactobacillus pasteurii*, CL: *Clostridium luticellarii*, LP: *Limnochorda pilosa*, PM: *Phocea massiliensis*, FC: *Fermentimonas caenicola*, PA: *Proteiniphilum acetatigenes*, AS: *Anaeromassilibacillus senegalensis*, TA: *Tepidanaerobacter acetatoxydans*, CLL: *Clostridium limosum*, CJ: *Clostridium jeddahense*, CHM: *Christensenella massiliensis*, LA: *Lactobacillus acetotolerans*, AM: *Ardenticatena maritima, SS: Syntrophaceticus schinkii*, PP: *Pelomonas puraquae*, AR: *Atopobium rimae*

Next, redundancy analysis (RDA) correlations were assessed to evaluate the relationship between different bacterial genera and the chemical properties of samples collected from different layers of pit mud (Fig. 2). The first two axes in this analysis accounted for 86.10% of the observed variation in bacterial communities, consistent with a significant correlation between bacterial communities and chemical properties. Pit mud samples collected from different spatial positions also exhibited clear separation from one another in this RDA analysis, consistent with the marked heterogeneity of the local ecosystem in which Chinese strong-flavor liquor is brewed. As shown in Fig. 2, LA (*Lactobacillus acetotolerans*), AS (*Anaeromassilibacillus senegalensis*), CK (*Clostridium kluyveri*), CL (*Clostridium luticellarii*), PRS (*Proteiniphilum saccharofermentans*), PS (*Petrimonas sulfuriphila*), and CLL (*Clostridium limosum*), which were all found in samples from the lower layer of the pit wall, were strongly positively correlated with total carbon, humus, K^+^, NH_4_^+^-N, Mg^2+^, and PO_4_^3−^, whereas CJ (*Clostridium jeddahense*), HS (*Hydrogenoanaerobacterium saccharovorans*), SH (*Sedimentibacter hydroxybenzoicus*), PEM (*Petrimonas mucosa*), AM (*Ardenticatena maritima*), SS (*Syntrophaceticus schinkii*), CS (*Clostridium sporosphaeroides*), FC (*Fermentimonas caenicola*), and JL (*Janthinobacterium lividum*) were moderately positively correlated with these same chemical properties (Additional file 1).

### Correlations between bacterial communities and volatile compounds

**Fig. 3:**
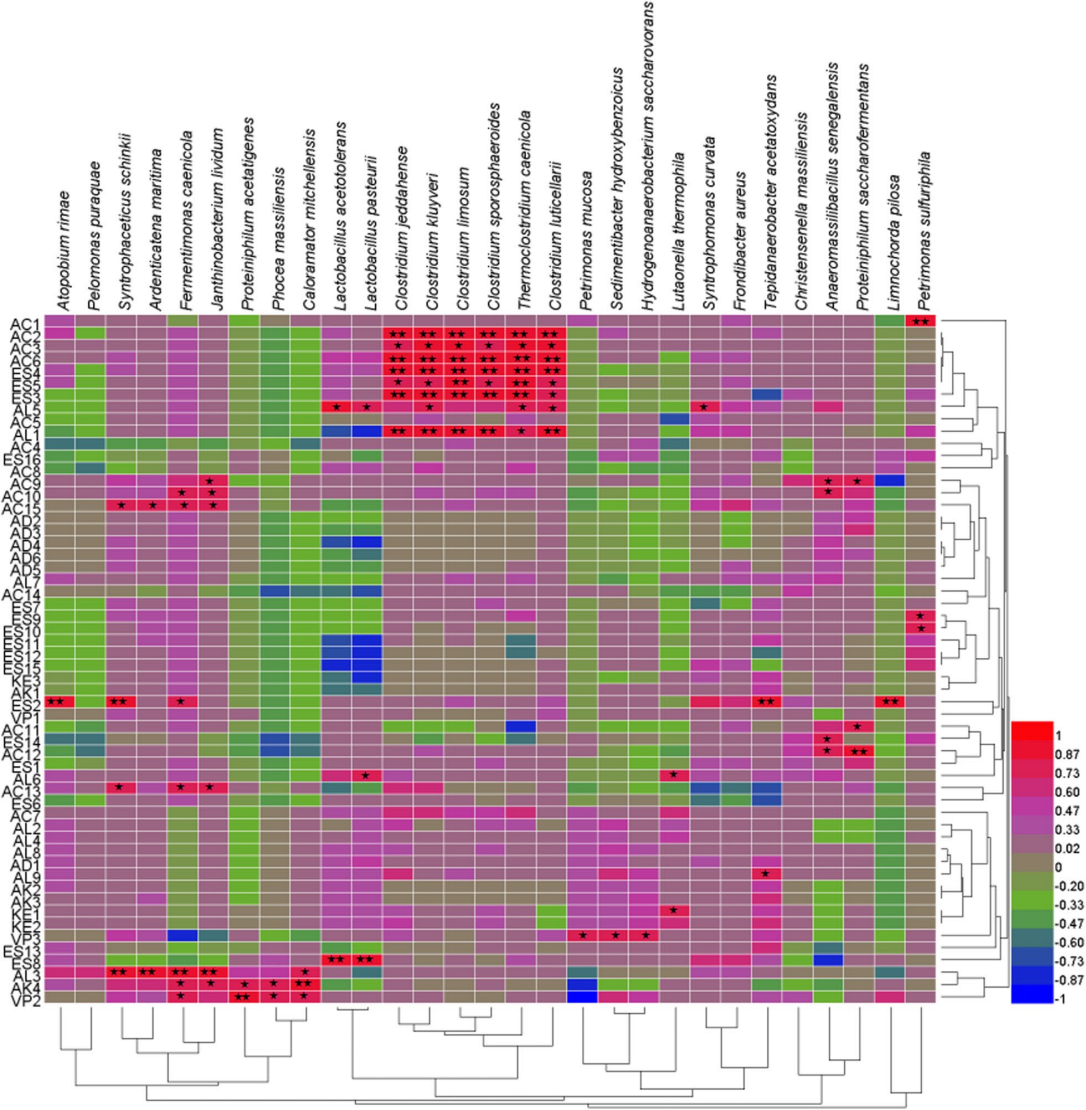
Analysis of correlations between bacterial diversity and volatile compound content in different pit mud layers. *P < 0.05, **P < 0.01

The correlative relationships between bacterial abundance and specific volatile compounds in different pit mud layers were next analyzed, revealing several correlations between specific pairs of bacteria and volatile compounds (Fig. 3).

AC1 (Propionic acid) was highly correlated with *Petrimonas sulfuriphila* abundance, while AC2 (Butyric acid), AC3 (Pentanoic acid), and AC6 (Caproic acid) were strongly positively correlated with the abundance of members of the *Clostridium* genus (*Clostridium jeddahense*, *Clostridium kluyveri*, *Thermoclostridium caenicola*, *Clostridium sporosphaeroides, Clostridium limosum*, *Clostridium luticellarii*). AC9 (Nonanoic acid) levels were significantly correlated with the abundance of *Janthinobacterium lividum, Anaeromassilibacillus senegalensis*, and *Proteiniphilum saccharofermentans*, whereas AC10 (n-Decanoic acid) levels were positively correlated with the abundance of *Fermentimonas caenicola*, *Janthinobacterium lividum*, and *Anaeromassilibacillus senegalensis.* Levels of AC11 (Benzoic acid) were also positively correlated with *Proteiniphilum saccharofermentans* abundance, and a positive correlation was observed between AC12 (Decanoic acid) and both *Anaeromassilibacillus senegalensis* and *Proteiniphilum saccharofermentans.* AC13 (Benzeneacetic acid) was positively correlated with *Syntrophaceticus schinkii*, *Fermentimonas caenicola*, and *Janthinobacterium lividum.* In addition, AC15 (n-Hexadecanoic acid) was positively correlated with *Syntrophaceticus schinkii*, *Ardenticatena maritima*, *Fermentimonas caenicola*, and *Janthinobacterium lividum.*

With respect to esters, ES2 (Ethyl acetate) levels were closely related to the abundance of *Atopobium rimae*, *Syntrophaceticus schinkii*, *Fermentimonas caenicola*, *Tepidanaerobacter acetatoxydans*, and *Limnochorda pilosa*, while the levels of ES3 (Ethyl caproate), ES4 (Ethyl butyrate), and ES5 (Ethyl heptanoate) all displayed positively related to the abundance of members of the *Clostridium* genus. ES8 (Ethyl lactate) levels were closely related to the abundance of *Lactobacillus pasteurii* and *Lactobacillus acetotolerans.* Both ES9 (Ethyl caprate) and ES10 (Ethyl undecanoate) levels were positively correlated with *Petrimonas sulfuriphila*, whereas ES14 (Ethyl phenylacetate) was significantly positively correlated with *Anaeromassilibacillus senegalensis.*

AL1 (Hexanol) levels were significantly correlated with the abundance of *Clostridium jeddahense*, *Clostridium kluyveri*, *Thermoclostridium caenicola*, *Clostridium sporosphaeroides, Clostridium limosum*, and *Clostridium luticellarii.* AL3 (Isobutanol) was significantly related to *Syntrophaceticus schinkii*, *Ardenticatena maritima, Janthinobacterium lividum*, *Fermentimonas caenicola*, and *Caloramator mitchellensis* abundance. AL5 (1-Butanol) was closely associated with *Lactobacillus pasteurii*, *Lactobacillus acetotolerans, Clostridium kluyveri*, *Thermoclostridium caenicola*, *Clostridium luticellarii*, and *Syntrophomonas curvata*. AL6 (2,3-butanediol) levels were significantly positively correlated with the abundance of *Lactobacillus pasteurii* and *Lutaonella thermophila*, while AL9 (2,3-butanediol) was positively correlated with *Tepidanaerobacter acetatoxydans.*

KE1 (2-octanone) levels were positively correlated with *Lutaonella thermophila* abundance, while AK4 (2-phenyl-2-butenal) was significantly positively correlated with the abundance of *Fermentimonas caenicola*, *Janthinobacterium lividum*, *Proteiniphilum acetatigenes*, *Phocea massiliensis*, and *Caloramator mitchellensis.* VP2 (2-Methylphenol) levels were also significantly correlated with pit mud *Fermentimonas caenicola*, *Proteiniphilum acetatigenes*, *Phocea massiliensis*, and *Caloramator mitchellensis* abundance, whereas, VP3 (3-methylphenol) levels were closely associated with the abundance of *Petrimonas mucosa, Sedimentibacter hydroxybenzoicus*, and *Hydrogenoanaerobacterium saccharovorans.*

## Discussion

Chinese strong-flavor liquor production is characterized by its fermentation in a purpose-built pit (~ 3.3 m long, 2.0 m wide, and 2.5 m deep). The bottom and walls of this pit are covered with a microbe-rich type of clay known as pit mud, with the nutrient composition of this pit mud generally being regarded as being critical to the composition, stability, and evolution of the microbial community found therein (Wu et al. 2022). The most critical physicochemical characteristics of pit mud include moisture, pH, NH_4_^+^-N, total carbon, humus, PO_4_^3−^, K^+^, Mg^2+^, and Ca^2+^ content, as all of these have the potential to shape the growth and metabolic activity of local microbial populations and to thereby shape the overall community structure of the pit mud microflora. Prior studies have demonstrated correlations between pit mud quality and certain properties such as pH, NH_4_^+^, available phosphorus (PO_4_^3−^), and Ca^2+^, with pH, NH_4_^+^ and PO_4_^3−^ levels being positively correlated with pit mud quality, whereas Ca^2+^ levels were negatively correlated with such quality (Zhang et al. 2020). The results of this study further revealed that the physicochemical properties of pit mud differ in the different layers within the fermentation pit. Specifically, the highest levels of NH_4_^+^-N, total carbon, humus, Mg^2+^, and PO_4_^3−^ were observed in the mud from the lower layer of the pit wall, followed by the mud from the bottom layer of the pit. K^+^ levels in the bottom layer of the pit were more similar to those in the lower layer of the pit wall and significantly increased relative to other pit mud layers. Maximum moisture, pH, and Ca^2+^ levels were observed in the bottom pit mud layer. Together, these results thus reaffirm that pit mud physicochemical properties exhibit spatially defined variations (Additional file 2).

Li et al. (2019) previously found that the physicochemical variability present in different pit mud layers can contribute to differences in the composition of the local microbial community, in line with the results of the present study. For example, *Lactobacillus acetotolerans*, *Anaeromassilibacillus senegalensis*, *Proteiniphilum saccharofermentans*, *Petrimonas sulfuriphila*, *Clostridium kluyveri*, and *Clostridium luticellarii*, all of which were located in the lower wall pit mud layer, were positively correlated with total carbon, humus, K^+^, NH_4_^+^-N, Mg^2+^, and PO_4_^3−^ (Fig. 2), whereas the abundance of *Christensenella massiliensis*, which was primarily located in the upper wall layer, was negatively correlated with NH_4_^+^-N, total carbon, humus, and PO_4_^3−^ levels (Fig. 2). This study further revealed that members of the *Clostridium* genus, which is a key functional genus in pit mud, were present at particularly high levels in the bottom and lower wall pit mud layers. This may explain the observation that the lower *Zaopei* layer yields better quality Chinese strong-flavor liquor relative to the middle and upper *Zaopei* layers.

In addition to serving as a critical habitat for the microbes that facilitate the fermentation of Chinese liquor, pit mud-derived microbes are also responsible for the production of volatile flavoring compounds (Hu et al. 2016). Zhang et al. (2021) found that the production of these volatile compounds in the context of fermentation was the result of a series of metabolic reactions that were impacted by complex microbial community dynamics. Dominant microbial species present in pit mud samples have previously been shown to include various species of *Clostridium*, *Lactobacillus*, *Bacillus*, and methanogens (Ding et al. 2014). *Clostridium* species are generally regarded as the primary functional bacteria in the pit mud, and their abundance is generally much higher in older pit mud relative to newly prepared pit mud, thus shaping overall pit mud quality (Hu et al. 2015). In this study, *Clostridium* abundance was detected at high levels in the lower layer of the pit mud wall wherein these microbes were found to play an important role in the production of volatile aroma and flavor compounds. Indeed, strong correlations were observed between *Clostridium* abundance and the levels of caproic acid, ethyl caproate, ethyl butyrate, and hexanol in pit mud samples, in addition to a moderate correlation with butyric acid levels, with all of these compounds being important aromatic components present within Chinese strong-flavor liquor. Lactic acid bacteria are another group of important pit mud microorganisms that influence overall pit mud quality. In this study, *Lactobacillus* species were present primarily in the upper, middle, and bottom layers of pit mud (Fig. 1). The lactic acid produced by these bacteria can be used to synthesize ethyl lactate, which is a key flavoring compound in Chinese strong-flavor liquor (Gao et al. 2021). Most lactobacilli produce large quantities of lactic acid, which can also serve as a precursor for lactic acetate production. Excess lactic acid production, however, can drive ferrous lactate and calcium lactate formation, with these compounds ultimately contributing to pit mud degeneration (Hu et al. 2021). *Lactobacillus* abundance was found to be positively correlated with ethyl lactate levels (Fig. 3). Moreover, *Petrimonas sulfuriphila* abundance was closely associated with propionic acid levels, *Syntrophomonas curvata* was related with 1-butanol levels, *Proteiniphilum acetatigenes* abundance was significantly correlated with 2-methylphenol levels, *Hydrogenoanaerobacterium saccharovorans* abundance was closely associated with 3-methylphenol levels, and a significant positive correlation was observed between *Caloramator mitchellensis* abundance and levels of 2-phenyl-2-butenal.

Pit mud quality is a key determinant of the quality and flavor of the liquor fermented therein, with such quality being a result of the physicochemical properties of the pit mud as well as the flavor compounds and microbial species found therein. Here, a PCR-DGGE approach was used to analyze the microbial community structure in pit mud samples collected from different locations, with physicochemical properties and flavor compounds in these samples additionally being analyzed. Subsequent correlative analyses of these three factors revealed pit mud samples from the lower wall of the fermentation pit to contain higher levels of available phosphorus (PO_4_^3−^), available potassium (K^+^), total carbon, humus, and Mg^2+^ relative to other analyzed samples. Moreover, the pH of the pit mud samples from this layer was close to neutral, which may be conducive to the metabolic activity of the functional bacteria in this layer. As such, the lower wall layer of pit mud was found to be of the highest quality, followed by the bottom pit mud layer. Studies of the volatile compounds found within pit mud further revealed that the lower wall pit mud layer contained the highest total levels of acids, esters, alcohols, and aldehydes. Together these data offer new insight regarding the mechanisms underlying volatile compound accumulation in pit mud in the context of Chinese strong-flavor liquor production, providing a foundation for future efforts to improve and maintain pit mud quality to enhance this fermentation process.

## Supplementary Information

**Additional file 1. **Nucleotide sequences of the bands.

**Additional file 2: Table S1.** Microorganism gray values of denaturing gradient gel electrophoresis (DGGE) gels in pit mud samples collected from different positions within the fermentation pit. Lanes U, M, D, and B respectively correspond to pit mud samples from the upper wall, middle wall, lower wall, and bottom layers of the cellar.

## Data Availability

Please contact author for data requests.
