# Supplementary material for: Bacterial diversity associated with volatile compound accumulation in pit mud of Chinese strong-flavor baijiu pit
Source: AMB Express. 2023 Jan 7;13:3. doi: 10.1186/s13568-023-01508-z (PMC9825687; doi:10.1186/s13568-023-01508-z)
Supplement: Supplementary file 1 — Additional file 1. Nucleotide sequences of the bands. [file 13568_2023_1508_MOESM1_ESM.doc]

**Supplementary materials**

**Additional file1: Nucleotide sequences of the bands**

Band 1: ATTACCGCGGCTGCTGGCACGTAGTTAGCCGGGGCTTTCTGGCAGGGTACCGTCACTTCCTTCGTCCCCTGCAACAGGGTTTTACGACCCGAAGGCCTTCGTCACCCACGCGGCGTCGCTGCGTCAGGCTTTCGCCAATTGCGCAATATTCCCCACTGCTGCCTCCCGTAGG

Band 2:

CCTACGGGAGGCAGCAGTGGGGAATATTGCGCAATGGGGGAAACCCTGACGCAGCAACGCCGCGTGAGTGAAGAAGGCCTTCGGGTCGTAAAGCTCTGTCATAGCCGACGAAGAATGACTGTAAGCTATGAGGAAGCCACGGCTAACTACGTGCCAGCAGCCGCGGTAAT

Band 3:

ATTACCGCGGCTGCTGGCACGTAGTTAGCCGGTGCTTATTCTTCAGGTACCGTCATTAGCAAGAGATATTAGCTCTCACCGTTTCTTCCCTGACAAAAGAGCTTTACAACCCGAAGGCCTTCTTCACTCACGCGGCATTGCTGGATCAGGCTTTCGCCCATTGTCCAAAATTCCCCACTGCTGCCTCCCGTAGG

Band 4:

CCTACGGGAGGCAGCAGTGGGGAATATTGCACAATGGGGGAAACCCTGATGCAGCGACGCCGCGTGAAGGATGAAGTATTTCGGTATGTAAACTTCTATCAGTAGGGAAGAAAATGACGGTACCTGACTAAGAAGCCCCGGCTAACTACGTGCCAGCAGCCGCGGTAAT

Band 5:

ATTACCGCGGCTGCTGGCACGTAGTTAGCCGGGGCTTCCTCTTCGGGTACTGTCCTTTTCATCCCCGATGACTGAGCTTTACAACCCGAAGGCCTTCTTCGCTCACGCGGCGTCGCTGCGTCAGGGTTGCCCCCATTGCGCAATATTCCCCACTGCTGCCTCCCGTAGG

Band 6:

CCTACGGGAGGCAGCAGTGAGGRATATTGGWCAATGGGCGGAAGCCTGAACCAGCCATGCCGCGTGCAGGAAGACGGCCTTATGGGTTGTAAACTGCTTTTATGTGAGGATAATATTCATCTTGCGAGGTGGAATGAAGGTATCACATGAATAAGGATCGGCTAACTCCGTGCCAGCAGCCGCG

GTAAT

Band 7:

ATTACCGCGGCTGCTGGCACGTAGTTAGCCGTGGCTTCCTCCTCCGGTACCGTCATTATCGTCCCGGAAGACAGAGCTTTACAATCCGAAAACCTTCTTCACTCACGCGGCGTTGCTGCATCAGGGTTTCCCCCATTGTGCAATATGCCCCACTGCTGCCTCCCGTAGG

Band 8:

ATTACCGCGGCTGCTGGCACGGAGTTAGCCGATCCTTATTCGCAAGGTACATGCAAAGGAGGACACGTCCCCCACTTTATTCCCTTGCAAAAGAAGTTTACAATCCATAAGACCTTCATCCTTCACGCGACTTGGCTGGTTCAGAGTTGCCTCCATTGACCAATATTCCTCACTGCTGCCTCCCGTAGG

Band 9:

CCTACGGGAGGCAGCAGTGAGGGATATTGGACAATGGATGGAAATCTGATCCAGCCATGCCGCGTGCAGGAAGACGGCCCTATGGGTTGTAAACTGCTTTTATACGGGAGCAATAAGATCTACGCGTAGACTGATGAGAGTACCGTATGAATAAGCATCGGCTAACTCCGTGCCAGCAGCCGCGGTAAT

Band 10:

ATTACCGCGGCTGCTGGCACGTAGTTAGCCGGAGCTTCCTCCTCAGGTACCGTCATTTTCGTCCCTGAGGACAGAGGTTTACAATCCAGAGGACCGTCTTCCCTCACGCGGCGTCGCTGCATCAGGCTTTCGCCCATTGTGCAATATCCCTCACTGCTGCCTCCCGTAGG

Band 11:

ATTACCGCGGCTGCTGGCACGTAGTTAGCCGTGGCTTGTTCCTCGGGTACCGTCATTTCCGTCCCCGATCAAAGAAGTTTACGACCCGAAAGCCTTCATCCTTCACGCGGCGTTGCTGCGTCAGGGTTTCCCCCATTGCGCAATATTCCCCACTGCTGCCTCCCGTAGG

Band 12:

CCTACGGGAGGCAGCAGTGGGGGATATTGCACAATGGAGGAAACTCTGATGCAGCAACGCCGCGTGAGGGAAGACGGTCTTCGGATTGTAAACCTTTGTCCTTGGTGAAGATAATGACGGTAGCCGAGGAGGAAGCTCCGGCTAACTACGTGCCAGCAGCCGCGGTAAT

Band 13:

CCTACGGGAGGCAGCAGTGGGGAATATTGCACAATGGAGGGAACTCTGATGCAGCGACGCCGCGTGAGTGAAGAAGGTTTTCGGATCGTAAAACTCTGTCCTTGGTGACGAAAGGAATGACGGTAGCCAAGGAGGAAGCCCCGGCTAACTACGTGCCAGCAGCCGCGGTAAT

Band 14:

ATTACCGCGGCTGCTGGCACGTAGTTAGCCGGGGCTTGCTGGTTAGGTACCGTCAATTCATATCACTGTTCGCAATATGACTCTTCGTCCCTTACAACAGAGCTTTACGACCCTAAGGCCTTCTTCGCTCACGCGGCGTCGCTGCGTCAGGGTTTCCCCCATTGCGCAGTATTCCCCACTGCTGCCTCCCGTAGG

Band 15:

CCTACGGGAGGCAGCAGTGAGGAATATTGGTCAATGGAGGCAACTCTGAACCAGCCAAGTCGCGTGAAGGATGAAGGTCTTATGGATTGTAAACTTCTTTTGCAAGGGAATAAAGTGGGGGACGTGTCCTCCTTCGCATGTACCTTGCGAATAAGGATCGGCTAACTCCGTGCCAGCAGCCGCGGTAAT

Band 16:

ATTACCGCGGCTGCTGGCACGGAGTTAGCCGATCCTTATTCGCAAGGTACATGCAAAGGAAGACACGTCCCCCACTTTATTCCCTTGCAAAAGAAGTTTACAACCCGTAGGGCCGTCTTCCTTCACGCGACGTGGCTGGTTCAGACTTGCGTCCATTGACCAATATTCCTCACTGCTGCCTCCC

GTAGG

Band 17:

ATTACCGCGGCTGCTGGCACGCTAGTTAGCCGTGACTTTCTGGTTGATTACCGGCAAATAATGAGCAGTTACTCTCACTATCTTTCTTCACCAACAACAGAGCTTTACGATCCGAAAACCTTCTCCACTCACGCGGCGTTGCTCCATCAGACTTGCGTCCATTGTGGAAGATTCCCTACTGCTGCCTCCCGTAGG

Band 18:

CCTACGGGAGGCAGCAGTAGGGAATCTTCCACAATGGACGCAAGTCTGATGGAGCAACGCCGCGTGAGTGAAGAAGGTTTTCGGATCGTAAAGCTCTGTTGTTGGTGAAGAAAGATAGTGAGAGTAACTGCTCATTATTTGCCGGTAATCAACCAGAAAGTCACGGCTAACTACGTGCCAGCAGCCGCGG

TAAT

Band 19:

ATTACCGCGGCTGCTGGCACGTAGTTAGCCGGGGCTTCCTCCGGGGGTACCGTCCGTTCTCGTCCCCCCAGACAGAGGTTTACAACCCGAAGGCCTTCGTCCCTCACGCGGCGTCGCTCGGTCAGGCTTTCGCCCATTGCCGAAAATTCCCTACTGCTGCCTCCCGTAGG

Band 20:

ATTACCGCGGCTGCTGGCACGTAGTTAGCCGGAGCTTGCTGCCTAGGTACCGTCATTATCGTCCCTAGGTACAAAAGTTTACAATCCGAAGACCGTCTTCCTTCACGCGGCGTTGCTGCATCAGAGTTTCCTCCATTGTGCAATATCCCCCACTGCTGCCTCCCGTAGG

Band 21:

CCTACGGGAGGCAGCAGTGAGGAATATTGGTCAATGGACGCAAGCCTGAACCAGCCACGTCGCGTGAAGGAAGACGGCCCTACGGGTTGTAAACTTCTTTTGTAAGGGAATAAAGTGAGTCACGCGTGACTTTTTGCATGTACCTTACGAATAAGGATCGGCTAACTCCGTGCCAGCAGCCGCG

GTAAT

Band 22:

ATTACCGCGGCTGCTGGCACGGAGTTAGCCGATCCTTATTCGTAAGGTACATGCAAAAAGTCACGCGTGACTCACTTTATTCCCTTACAAAAGAAGTTTACAACCCGTAGGGCCGTCTTCCTTCACGCGACGTGGCTGGTTCAGACTTGCGTCCATTGACCAATATTCCTCACTGCTGCCT

CCCGTAGG

Band 23:

CCTACGGGAGGCAGCAGTGGGGGATATTGCACAATGGAGGAAACTCTGATGCAGCAACGCCGCGTGAAGGAAGACGGTCTTCGGATTGTAAACTTTTGTACCTAGGGACGATAATGACGGTACCTAGGCAGCAAGCTCCGGCTAACTACGTGCCAGCAGCCGCGGTAAT

Band 24:

ATTACCGCGGCTGCTGGCACGTAGTTAGCCGTGGCTTCCTCCTCCGGTACCGTCATTATCGTCCCGGAAGRCAGAGCTTTACAATCCGAAAACCTTCATCACTCACGCGGCGTTGCTCGGTCAGGCTTGCGCCCATTGCCGAAGATTCCCAACTGCTGCCTCCCGTAGG

Band 25:

ATTACCGCGGCTGCTGGCACGTAGTTAGCCGGAGCTTCCTCCTCGGCTACCGTCATTATCGGCACCGGGGACAGAGGTTTACAATCCGAAAACCGTCTTCCCTCACGCGGCGTCGCTGCATCAGAGTTCCCTCCATTGTGCAAGATCCCCCACTGCTGCCTCCCGTAGG

Band 26:

ATTACCGCGGCTGCTGGCACGTAGTTAGCCGGGGCTTCCTCCCATGGTACCGTCACTCCCTTCGTCCCATGGGACAGAGGTTTACAATCCGAAAACCTTCTTCCCTCACGCGGCGTTGCTGGGTCAGGCTTTCGCCCATTGCCCAATATTCCCCACTGCTGCCTCCCGTAGG

Band 27:

CCTACGGGAGGCAGCAGTGGGGAATATTGCACAATGGGCGGAAGCCTGATGCAGCAACGCCGCGTGAGTGAAGAAGGTTTTCGGATTGTAAAGCTCTGTCATATGGGACGATAGTGACGGTACCATATGAGGAAGCCACGGCTAACTACGTGCCAGCAGCCGCGGTAAT

Band 28:

ATTACCGCGGCTGCTGGCACGTAGTTAGCCGGGACTTATTCCTGGGGTACCGTCCTTCCTCTTCCCCCAGAAAAGCGGTTTACGACCCGAAGGCCTTCTTCCCGCACGCGATGTCGCTGCATCAGGGTTGCCCCCATTGTGCAAGATTCCTCACTGCTGCCTCCCGTAGG

Band 29:

ATTACCGCGGCTGCTGGCACGTAGTTAGCCGGGGCTTCCTCGCAGGGTACCGTCACTTCCTTCGTCCCCTGCAACAGGGCTTTACGACCCGAAGGCCTTCGTCACCCACGCGGCGTCGCTGCGTCAGGCTTTCGCCCATTGCGCAAAATTCCCCACTGCTGCCTCCCGTAGG
