# Supplementary material for: Bacterial diversity associated with volatile compound accumulation in pit mud of Chinese strong-flavor baijiu pit
Source: AMB Express. 2023 Jan 7;13:3. doi: 10.1186/s13568-023-01508-z (PMC9825687; doi:10.1186/s13568-023-01508-z)
Supplement: Supplementary file 2 — Additional file 2: Table S1. Microorganism gray values of denaturing gradient gel electrophoresis (DGGE) gels in pit mud samples collected from different positions within the fermentation pit. Lanes U, M, D, and B respectively correspond to pit mud samples from the upper wall, middle wall, lower wall, and bottom layers of the cellar. [file 13568_2023_1508_MOESM2_ESM.docx]

**Additional file 2:**

Table S1 Microorganism gray values of denaturing gradient gel electrophoresis (DGGE) gels in pit mud samples collected from different positions within the fermentation pit. Lanes U, M, D, and B respectively correspond to pit mud samples from the upper wall, middle wall, lower wall, and bottom layers of the cellar.

| Closest relative (NCBI accession no.) | U | M | D | B |
| --- | --- | --- | --- | --- |
| *Caloramator mitchellensis* (NR_117542.1 ) | 0 | 159.708±7.48 | 0 | 0 |
| *Janthinobacterium lividum* (NR_026365.1) | 0 | 162.393±5.12 | 86.751±2.34 | 0 |
| *Tepidanaerobacter acetatoxydans* (NR_074537.1) | 152.56±4.47 | 0 | 0 | 0 |
| *Frondibacter aureus* (NR_134733.1) | 162.5±5.32 | 0 | 0 | 130.6±2.33 |
| *Syntrophomonas curvata* (NR_025752.1) | 127.5±4.35 | 0 | 0 | 116.5±2.11 |
| *Petrimonas sulfuriphila* (NR_042987.1) | 0 | 0 | 163.774±4.45 | 120.685±3.21 |
| *Lutaonella thermophila* (NR_044451.1) | 126.789±2.11 | 0 | 0 | 0 |
| *Hydrogenoanaerobacterium saccharovorans* (NR_044425.1) | 120.529±2.22 | 0 | 110.02±2.09 | 0 |
| *Thermoclostridium caenicola* (NR_126170.1) | 208.405±3.12 | 0 | 0 | 0 |
| *Clostridium sporosphaeroides* (NR_044835.2) | 152.202±3.33 | 165.012±4.53 | 158.946±5.76 | 150.953±2.49 |
| *Sedimentibacter hydroxybenzoicus* (NR_029146.1) | 138.101±4.21 | 0 | 133.25±3.65 | 0 |
| *Clostridium kluyveri* (NR_074447.1) | 0 | 0 | 222.607±2.88 | 161.255±3.46 |
| *Petrimonas mucosa* (NR_148808.1) | 231.844±5.57 | 0 | 231.113±2.89 | 211.595±3.17 |
| *Proteiniphilum saccharofermentans* (NR_148807.1) | 0 | 0 | 168.043±5.12 | 0 |
| *Lactobacillus pasteurii* (NR_117058.1) | 313.202±3.35 | 235.833±2.55 | 0 | 296.953±4.46 |
| *Clostridium luticellarii* (NR_145907.1) | 0 | 0 | 205.596±4.56 | 0 |
| *Limnochorda pilosa* (NR_136767.1) | 280.202±2.89 | 286.833±2.78 | 0 | 289.953±3.45 |
| *Phocea massiliensis* (NR_144748.1) | 146.763±2.41 | 185.809±2.21 | 0 | 170.65±4.23 |
| *Fermentimonas caenicola* (NR_148809.1) | 80.65±1.46 | 221.304±2.65 | 181.953±2.43 | 164.607±3.65 |
| *Proteiniphilum acetatigenes* (NR_043154.1) | 0 | 173.568±2.98 | 0 | 161.257±3.43 |
| *Anaeromassilibacillus senegalensis* (NR_144727.1) | 0 | 0 | 154.856±2.89 | 0 |
| *Hathewaya histolytica* (NR_104889.1) | 162.214±3.32 | 0 | 0 | 0 |
| *Clostridium jeddahense* (NR_144697.1) | 183.237±3.87 | 178.833±2.37 | 168.619±3.68 | 175.458±5.03 |
| *Christensenella massiliensis* (NR_144742.1) | 154.355±2.05 | 0 | 175.547±4.45 | 0 |
| *Lactobacillus acetotolerans* (NR_044699.2) | 0 | 0 | 158.339±2.77 | 0 |
| *Ardenticatena maritima* (NR_113219.1) | 0 | 176.572±2.89 | 163.101±5.62 | 0 |
| *Syntrophaceticus schinkii* (NR_116297.1) | 0 | 171.562±3.76 | 151.63±4.45 | 0 |
| *Pelomonas puraquae* (JQ660112.1) | 0 | 165.352±3.76 | 0 | 0 |
